# Novel Conjugates of 1,3-Diacylglycerol and Lipoic Acid: Synthesis, DPPH Assay, and RP-LC-MS-APCI Analysis

**DOI:** 10.1155/2011/419809

**Published:** 2011-09-28

**Authors:** Samanthi R. P. Madawala, Rolf E. Andersson, Jelena A. Jastrebova, Maria Almeida, Paresh C. Dutta

**Affiliations:** ^1^Department of Food Science, Uppsala BioCenter, Swedish University of Agricultural Sciences (SLU), P.O. Box 7051, 750 07 Uppsala, Sweden; ^2^Department of Chemistry, Uppsala BioCenter, Swedish University of Agricultural Sciences (SLU), P.O. Box 7015, 750 07 Uppsala, Sweden; ^3^Aktivia Science Work AB, Karolinska Institute Science Park, Alfred Nobels Allé 10, 141 52 Huddinge, Sweden

## Abstract

1,3-Diacylglycerol is known to reduce body weight and fat deposits in humans. **α**-Lipoic acid is a potent antioxidant and effective against many pathological conditions, including obesity and related metabolic syndromes. The present work is based on the hypothesis that the hybrid molecules of 1,3-diacylglycerol and lipoic acid possess synergistic and/or additive effects compared with the parent compounds against obesity, overweight, and related metabolic syndromes. Laboratory scale synthesis of 1,3-dioleoyl-2-lipoyl-sn-glycerol (yield 80%) and 1,3-dioleoyl-2-dihydrolipoyl-sn-glycerol (yield 70%) was performed for the first time and supported by NMR and MS data. Free radical scavenging capacity of the conjugates was assayed using DPPH test. A remarkably high *in vitro* free radical scavenging capacity was demonstrated for the 1,3-dioleoyl-2-dihydrolipoyl-sn-glycerol (EC_50_ value 0.21). RP-HPLC-MS-APCI analysis showed satisfactory separation between the conjugates (R~1). Protonated molecular ion of the conjugates at *m/z* 809 and *m/z* at 811, respectively, and their characteristic fragment ions were abundant.

## 1. Introduction

As predicted by WHO, by 2015, approximately 2.3 billion adults will be overweight and more than 700 million will be obese [1]. The multitudinous pathological consequences of obesity and overweight include type II diabetes, oxidative stress, inflammation, cardiovascular diseases, hyperlipidemia, and hypertension. The emerging global public health issues of obesity, overweight, and related syndromes need to be addressed urgently with multiple strategies and approaches. 

**Figure 1:**
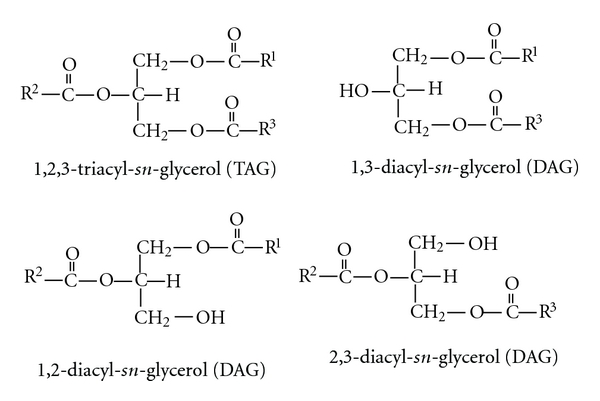
Chemical structures of triacylglycerol (TAG) and diacylglycerols (DAG) wherein R^1^, R^2^, and R^3^ are an alkyl or an alkenyl, hydrocarbon chain of a fatty acid (general formula R-COOH) esterified on the glycerol backbone.

The triacylglycerol (TAG) content in edible fats and oils generally exceeds 95%. Although a minor component, the levels of diacylglycerol (DAG) can be as much as ca. 10%, in some type of edible oils [2].  Figure 1 shows the chemical structures of TAG and DAG molecules. 1,3-DAG is mainly utilized by the body as a lean body mass energy source (or a direct or instant energy source) rather than being stored in the adipose tissue, in contrast to TAG, due to the different metabolic fates after absorption into the gastrointestinal epithelial cells [2]. Clinical studies with animals and humans on the effects of DAG oil have shown that it significantly decreases body weight, suppresses body fat accumulation, and lowers postprandial serum TAG levels, thus reducing obesity-related health risks [3, 4]. The mechanism causing the antiobesity effects of DAG is not clearly understood, and several pathways are suggested. After digestion, 1,3-DAG is converted into 1-(or 3-) monoacylglycerol (MAG), glycerol, and free fatty acids by the 1,3-lipases, in contrast to TAG, which produces 2-MAG and free fatty acids. In the epithelial cells of small intestines, 1- or 3-MAG is utilized poorly for the resynthesis of TAG because of the preference of DAG acyltransferase for the 2-MAG as a substrate. The free fatty acids generated from 1,3-DAG are shunted directly to the liver through the portal vein to be oxidized [2–4]. The increased *β*-oxidation results in decreased food intake by increasing satiety and subsequently reduces body weight [2, 5]. It is also suggested that a metabolite of 1,3-DAG is utilized which follows an *α*-glycerophosphate pathway, in contrast to TAG metabolites [6]. A serotonin-linked mechanism has also been proposed for DAG-mediated promotion of negative caloric balance, based on a study on Caco-2 cells with 1-MAG, a digestive product of DAG. Enhanced expression of genes associated with *β*-oxidation, fatty acid metabolism, and thermogenesis, in concomitance with an increase in the serotonin release from the Caco-2 cells, were reported [7]. The safety of dietary 1,3-DAG has been extensively studied, and it is considered to be as safe as other edible oils [8, 9]. 

Naturally occurring *α*-lipoic acid (LA) is the dextrorotatory isomer of 5-(dithiolane)-pentanoic acid [10]. The antioxidant properties of the LA and its reduced form dihydrolipoic acid (DHLA) system for scavenging of the reactive oxygen species (ROS), chelating metal ions, and regenerating cellular antioxidants have been extensively investigated [11, 12]. DHLA possesses superior antioxidant properties compared with LA. It has been suggested that both of these compounds are useful in the prevention and treatment of obesity and overweight, as well as a variety of pathological conditions linked to oxidative stress, for example, diabetes and cardiovascular disease, liver diseases, AIDS, and age-related disorders, [11–13]. LA has been in use as a supplement and is popularly known as the “metabolic antioxidant” or “the universal antioxidant” [11, 14]. 

The most important function of LA is as a cofactor of mitochondrial enzymes in energy metabolism. The redox pair LA/DHLA are highly effective against reactive oxygen species (ROS) and reactive nitrogen species (RNS). In addition to its antioxidant function by direct radical trapping and/or metal chelation, LA also modulates important signal transduction pathways that increase the endogenous cellular antioxidants, for example, glutathione (GSH), and lower inflammatory and antiapoptotic signalling pathways [15]. Supplementation of LA (800 mg/d) for four months showed significant body weight reduction: 8% in preobese and 9% in obese human subjects. The authors have suggested that LA may be an ideal candidate for treatment of obesity and related diseases [16]. It has been shown in rats that the reason for the considerable weight loss caused by LA acid is the suppression of hypothalamic adenosine monophosphate-activated protein kinase (AMPK) activity, resulting in enhanced energy expenditure. These effects were neither due to systemic toxicity nor dependent on leptin and its receptor [17]. Further, the authors found that LA increases glucose uptake and fatty acid oxidation by increasing AMPK in skeletal muscle, in contrast to its effects in the hypothalamus, and acts differently depending on the tissue types. Humans can tolerate several grams of LA administered orally without any adverse effect [18, 19]. 

The conventional antiobesity drugs generally have adverse side effects and are not effective in eliminating the main cause of obesity [20]. Extensive research is at present being conducted on low-energy diets and very-low-energy diets (VLEDs) in the management of body weight, obesity, and related pathological syndromes. Weight reduction by more than 15 kg can be achieved by VLED after 8 weeks, but maintaining this loss of weight during a longer period is difficult and that requires a combination of weight maintenance strategies [21]. A multitude of LA conjugates have been synthesized in order to enhance stability and the biological and functional properties of the parent components and/or to achieve dual functions [22–24]. 

This study is based on the hypothesis that the novel hybrids of DAG with LA and DHLA possess advantages compared with the parent compounds due to synergistic and/or cumulative effects. It is anticipated that these hybrid molecules can be used as functional food and/or novel ingredients and may open a new research area for their potential weight loss and antioxidant functions. In an acute pilot study in Wistar rats using a single dose of 200 mg/kg, both DOLA and DODHLA seemed to reduce food intake after 4 hours compared to control vehicle (unpublished results). The results prompted us to conduct the present study, and further feeding experiments are being pursued. As the first part of the study, we report here on the synthesis of 1,3-dioleoyl-2-lipoyl-*sn*-glycerol (DOLA) and 1,3-dioleoyl-2-dihydrolipoyl-*sn*-glycerol (DODHLA) hybrids [25]. In addition,* in vitro* DPPH-based free radical scavenging capacity and RP-HPLC-MS-APCI evaluations of the hybrid compounds are also presented. 

## 2. Materials and Methods

### 2.1. General Procedure and Materials

1,3-Dioleoyl-*sn*-glycerol from Nu-Check Prep, Inc., (Elysian, MN, USA) and LA were purchased from Sigma-Aldrich AB (Stockholm, Sweden). The chemicals and solvents, unless otherwise specified in the syntheses of the compounds in the examples, were purchased from VWR International AB (Stockholm, Sweden). The chromatographic separations were performed using silica gel (60 Å, 200–400 mesh). The compounds were analyzed by TLC: silica plates (Merck 60), and the compounds were visualized by treatment with a 10% solution of phosphomolybdic acid (PMA) in ethanol followed by heating. ^1^H, ^13^C NMR, and 2D experiments (COSY and HSQC-dept) spectra were obtained on a Bruker 400 MHz spectrometer (Bruker DRX, Germany), and chemical shifts (*δ*) are given in ppm relative to TMS. The spectra were recorded in CDCl_3_ as the solvent at 30°C.

### 2.2. Preparation of 1,3-Dioleoyl-2-lipoyl-sn-glycerol (**1**)

**Figure 2:**
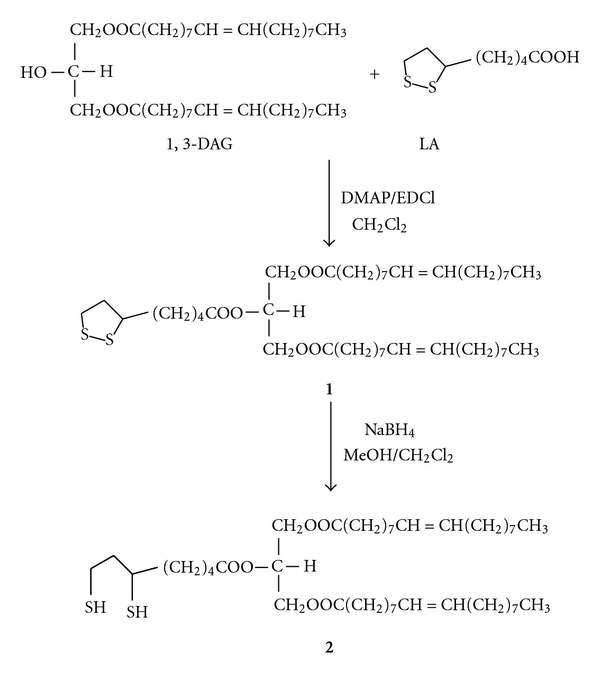
Scheme showing the structures and syntheses of 1,3-dioleoyl-2-lipoyl-*sn*-glycerol (DOLA, **1**) and 1,3-dioleoyl-2-dihydrolipoyl-*sn*-glycerol (DODHLA, **2**).

The synthesis of DOLA was performed following a published method with some modifications [26]. To a solution of DAG (1.61 mmol) in CH_2_Cl_2_ (9 mL) were added DMAP (42 mg, 0.34 mmol), LA (435 mg, 2.11 mmol), and EDCI (310 mg, 1.62 mmol) at 0°C with stirring. The reaction mixture was stirred at room temperature overnight. An extractive workup (CH_2_Cl_2_, dilute HCl, water, aqueous saturated NaCl) and drying (Na_2_SO_4_) of the combined organic extracts and concentration furnished the crude, which was purified by chromatography to give the pure compound, no trace of cis/trans isomerisation was observed by NMR analysis (^1^H, COSY, and HSQC-dept): 1,3-dioleoyl-2-lipoyl-*sn*-glycerol (**1**) (structure shown in Figure 2): (1 g, 80%). ^1^H NMR (CDCl_3_, 400 MHz), (*δ*) 5.45–5.30 (m, 4 H), 5.28–5.20 (m, 1 H), 4.35–4.25 (dd, 2 H), 4.18–4.10 (dd, 2 H), 3.60–3.50 (m, 1 H), 3.21–3.06 (m, 2 H), 2.50–2.40 (m, 1 H), 2.35–2.28 (m, 6 H), 2.05–1.96 (m, 8 H), 1.95–1.85 (m, 1 H), 1.75–1.56 (m, 8 H), 1.55–1.40 (m, 2 H), 1.39–1.20 (m, 40 H), 0.92–0.85 (t, 6 H). ^13^C NMR (CDCl_3_, 100 MHz), (*δ*) 173.2, 172.5, 130.0, 129.7, 69.1, 62.0, 56.3, 40.2, 38.5, 34.6, 34.0, 33.9, 31.9, 29.8, 29.7, 29.5, 29.4, 29.3, 29.2, 29.1, 29.0, 28.7, 27.3, 27.2, 24.8, 24.6, 22.7, 14.1.

### 2.3. Preparation of 1,3-Dioleoyl-2dihydrolipoyl-sn-glycerol (**2**)

The synthesis of DODHLA was conducted following a published method with some modifications [27]. DOLA (0.247 mmol) was dissolved in CH_2_Cl_2_/MeOH (1 : 5, v/v, 12 mL) under N_2_. NaBH_4_ (14 mg, 0.371 mmol) was added in portions, and the reaction mixture was stirred at room temperature under N_2_ (Figure 2). After about 2 h, aqueous 1 M HCl (5 mL) was added. An extractive workup (CH_2_Cl_2_, dilute HCl, water, aqueous saturated NaCl) and drying (Na_2_SO_4_) of the combined organic extracts and concentration furnished the crude, which was purified by chromatography to give the pure compound, no trace of *cis/trans* isomerisation was observed in NMR analysis: 1,3-dioleoyl-2Dihydrolipoyl-*sn*-glycerol (**2**): (140 mg, 70%). ^1^H NMR (CDCl_3_, 400 MHz), (*δ*) 5.43–5.35 (m, 4 H), 5.30–5.25 (m, 1 H), 4.36–4.25 (dd, 2 H), 4.20–4.12 (dd, 2 H), 3.01–2.90 (m, 1 H), 2.80–2.65 (m, 2 H), 2.40–2.30 (m, 6 H), 2.10–1.86 (m, 9 H), 1.80–1.45 (m, 11 H), 1.40–1.20 (m, 42 H), 0.95–0.86 (t, 6 H). ^13^C NMR (CDCl_3_, 100 MHz), (*δ*) 173.2, 172.5, 130.0, 129.7, 69.1, 62.0, 42.8, 39.3, 38.7, 34.2, 34.0, 31.9, 29.8, 29.7, 29.6, 29.5, 29.4, 29.3, 29.2, 29.1, 27.2, 27.1, 26.6, 24.8, 24.5, 22.7, 22.3, 14.1.

### 2.4. Evaluation of Free Radical Scavenging Capacity

**Figure 3:**
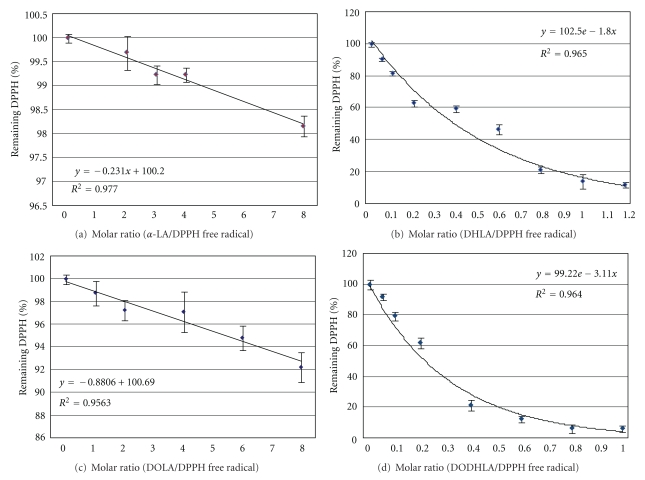
DPPH radical reduction (%) as a function of molar ratio of (a) LA/DPPH free radical; (b) DHLA/DPPH free radical; (c) DOLA/DPPH free radical; (d) DODHLA/DPPH free radical.

The *in vitro *free radical scavenging capacity of the test compounds was determined according to the DPPH (2,2Diphenyl-1-1picrylhydrazyl) method [28, 29]. Molar ratios between sample and DPPH in final mixture, from 1 to 8 in triplicates, were tested for *α*-LA and 1,-3-DOLA, while molar ratios from 0.05 to 1.2 were tested for DHLA and 1,3-DODHLA at room temperature along with a reagent blank. In each test 3.9 mL of 0.06 mM DPPH solution in toluene [30] was mixed with 0.1 mL of sample dissolved in toluene, and absorbance was measured at 515 nm at 15 min intervals until steady status was reached for the remaining DPPH percentage. Mean value of at least three replicates for each molar concentration along with error bars is presented in Table 1 and in Figure 3. Antiradical activity defined as the amount of antioxidant necessary to decrease the initial DPPH concentration by 50% (Efficient Concentration = EC_50_ (mol/L) and antiradical power (APR = 1/EC_50_)) [31] value was calculated.

### 2.5. Thin-Layer Chromatography (TLC)

The initial compounds used in the synthesis, LA, DO and the synthesized compounds DOLA and DODHLA were checked using TLC (Si, 0.25 mm). The optimal mobile phase mixture was hexane : diethyl ether : ethyl acetate: acetic acid (75 : 20 : 5 : 1, v/v/v/v). Approximately 20–30 *μ*g of the compounds were spotted. The plate was sprayed with PMA and visualized after drying in an oven at 120°C for 15 min.

### 2.6. RP-HPLC-MS-APCI

An HPLC-MS (HP 1100 Series, Agilent technologies Inc., Palo Alto, CA) equipped with a quaternary gradient pump, thermostated column compartment, thermostated autosampler, single quadrupole mass analyzer (G 1946D), Chemstation Rev.B.04.01 software. Chromatographic conditions were optimized based on the LC method [31]. DOLA and DODHLA were separated using a reversed phase Thermo Hypersil GOLD column, 150 × 4.6 mm i.d, 3 *μ*m particle size (Thermo Electron Corporation, UK). Mobile phase consisted of a tertiary mixture acetonitrile : 2-propanol : 5 mmol aqueous acetic acid (65 : 30 : 5, v/v/v) was used for column equilibration. Gradient elution was started with the same solvent mixture linearly changing to (25 : 70 : 5, v/v/v) over a period of 30 min. Flow rate was maintained at 0.6 mL/min, postrun equilibration time was 10 min, and column temperature was set at 20°C. Analytes were dissolved in hexane ca.1-2 *μ*g/*μ*L, and 1 *μ*L was injected. APCI was performed as follows; vaporizer temperature 300°C, drying gas flow rate 9 L/min and temperature 350°C, nebulizer pressure 60 psi, corona current 8 *μ*A, capillary voltage 3000 V and fragmentor voltage at 70 V. Total Ion Current (TIC) was recorded in SCAN mode at a mass range from *m/z* 100–1000.

## 3. Results

In Figure 2, the scheme on synthesis of the derivatives of 1,3-dioleoyl-*sn*-glycerol with LA and DHLA is shown. We have synthesized 1,3-DAG-lipoates only with oleic acid as a model in this study. Certain optimization during synthesis and preparative works using silica gel column chromatography were necessary. The chemical synthesis was supported with NMR data as shown in the methodology section.

Stable DPPH^●^ is considered as suitable for analysis performed at micromolar levels and for lipophilic compounds [28–30]. The free radical scavenging capacity of standard LA and DHLA was also evaluated with the same method to compare the reduction potential of the test hybrid compounds. The non-reduced compounds, LA and DOLA, showed a comparatively low interaction with DPPH^●^. LA did not interact with more than 98.2% of the initial DPPH^●^ at the highest tested molar ratio of 8, even after a longer time of more than 5 h. Similarly, at the same molar ratio, the results for DOLA showed that 92.3% of the DPPH^●^ remained unchanged, indicating a free radical scavenging activity that was slightly higher compared with LA (Figures 3(a) and 3(c)). Remarkably high free radical scavenging activity was observed for the reduced compounds DHLA and DODHLA. The time taken to reach the steady state varied from 20 min to 2.5 h, for the concentrations expressed as molar ratio 0.05–1.2 (Figures 3(b) and 3(d)). The interaction of the reduced compounds with DPPH^●^ was clearly dependent on both the concentration and the time, in contrast to the corresponding interaction of the nonreduced compounds. Similarly, the remaining DPPH^●^ measured for DHLA at molar ratio 1 was 14.4%, whereas that for DODHLA was 6.4%. The calculated EC_50_ value for DODHLA was 0.21, while that for DHLA was 0.38 (Table 1).

**Figure 4:**
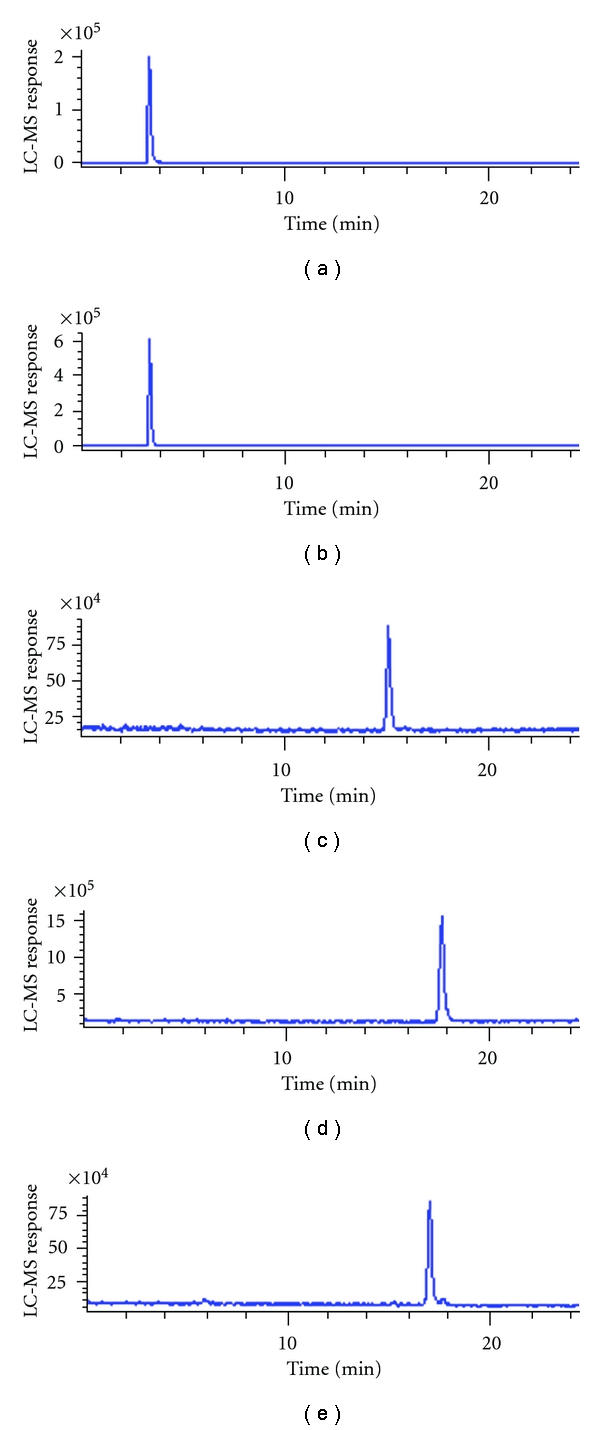
RP-HPLC-MS-APCI total ion and retention time of the test compounds. (a) LA; (b) DHLA; (c) DO; (d) DOLA; (e) DODHLA (Table 2 is referred for abbreviations).

**Figure 5:**
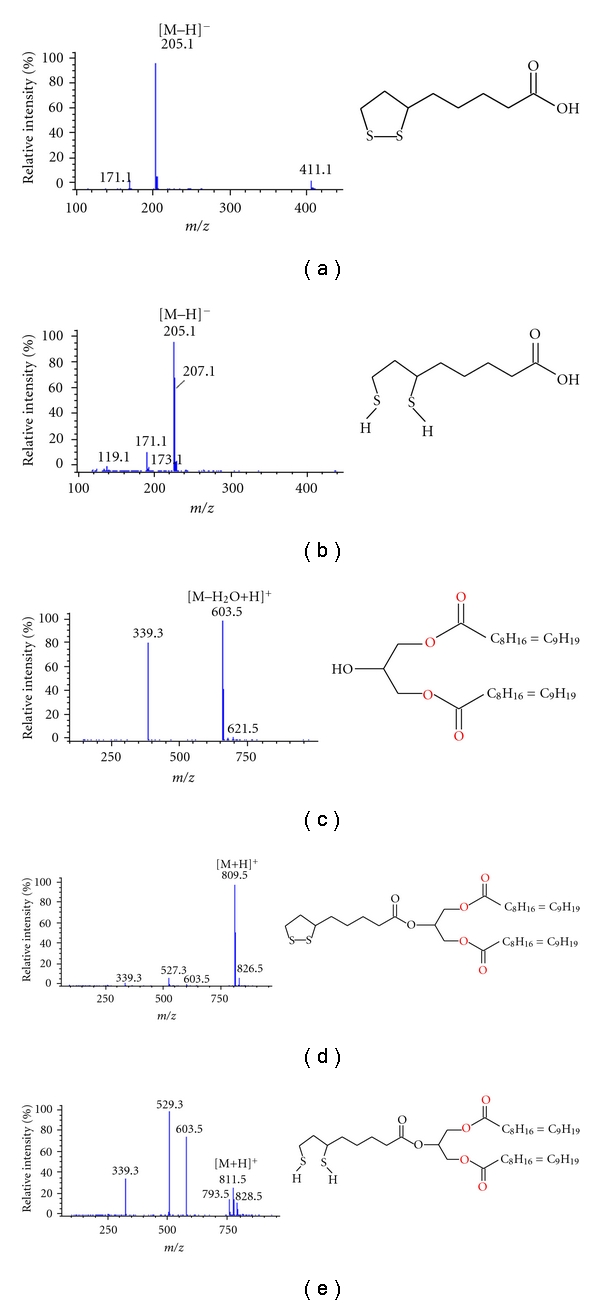
APCI mass spectra recorded at negative mode (a-b) [M-H]^−^ and recorded at positive ion mode (c–e) and the structures of the test compounds. (a) LA; (b) DHLA; (c) DO; (d) DOLA; (e) DODHLA (Table 2 is referred for abbreviations).

RP-HPLC-MS-APCI total ion chromatograms of the test compounds are shown in  Figures 4(a)–4(e). The elution order was related to the polarity of the compounds. The most polar LA and DHLA were eluted first followed by less polar DO, DODHLA, and DOLA. Satisfactory separation among the analyzed parent compounds and the conjugates except LA and DHLA were achieved (Table 2, Figure 4), and the calculated resolution between DOLA, and DODHLA was acceptable (R~1, results are not shown in Table). Due to their acidic properties, LA and DHLA could only be ionized in negative ion mode whereas DO, DOLA and DODHLA were easily ionized in positive ion mode. The APCI mass spectra, the major ions, and possible fragmentation pattern of each compound analysed are shown in Figures 5(a)–5(e) and Table 2. Deprotonated molecular ions [M–H]^−^ were the major ions observed for LA and DHLA with *m/z* 205 and 207, respectively. For DHLA the formation of [M–2H–H]^−^ ion was also observed at *m/z* 205.

In the mass spectrum for DO (Figure 5(c)) the major ion observed was at *m/z* 603 [M+H-18]^+^. Ion fragment at *m/z* 339 [M–RCOOH+H]^+^ showed dissociation of one oleic acid moiety in DO resulting protonated MAG. Protonated molecular ion at *m/z* 621 [M+H]^+^ was observed at very low abundance. A protonated molecular ion at *m/z* 809 [M+H]^+^ with minimum fragmentation was observed in DOLA (Figure 5(d)). Ion fragment at *m/z* 527 corresponding to one dissociated oleic acid [M–R_1_COOH+H]^+^ was observed. The ion fragment at *m/z* 339 [M–R_1_COOH–R_2_COOH + H_2_O]^+^ showed where an oleic acid and LA moiety were dissociated. A similar fragment ion was observed in the mass spectrum of DO albeit at much lower abundance. Ion fragment at *m/z* 603 [M–R_2_COOH+H]^+^ was the least abundant ion where only LA moiety was dissociated from the parent molecule. DODHLA showed the most fragmentation among all compounds tested where protonated molecular ion at *m/z *811 [M+H]^+^ appeared as only 40% of the base peak. This may suggest that DOLA is possibly more stable than DODHLA.

## 4. Discussion

1,3-DAG can be prepared with fatty acids from any fats, and oils and the fatty acid composition would depend on the substrate [32]. The physicochemical properties might vary depending on the acyl moieties within the synthesized DAG molecule. Fatty acids in 1,3-DAG are presumed to be utilized mainly for energy formation [2, 3]. It is common approach to synthesise new hybrid molecules with the anticipation of synergistic and/or dual effects due to their modified physical, chemical, and biological effects such as solubility and bioavailability, compared with the parent molecules [22, 24, 33, 34].

Studies have shown that obesity and metabolic syndrome-related pathological conditions such as hypertension, insulin resistance, diabetes, and hyperlipidemia, are linked with chronic inflammation and oxidative stress caused by the elevation of ROS and a lower antioxidant status. It is also well established that obese subjects have higher levels of oxidative stress biomarkers than leaner subjects [35, 36]. An antioxidant is generally defined as any substance in a low concentration, compared with a substrate, which significantly delays or prevents oxidation of the substrate. Moreover it alone or in conjunction with another component can act in different ways for example, it can chelate metal ions, reduce the effects of ROS through its free radical scavenging capacity, regenerate endogenous antioxidants, play a role in repairing systems, influence gene expression, and so forth, [18, 37–39]. The antioxidant activity of LA has been extensively studied. *In vitro* DPPH^●^ free radical activity of LA and some LA-coumarin derivatives dissolved in ethanol was studied previously [24]. We observed lower activity for LA tested in toluene compared to that study solvent may have affected the results. LA acts as an antioxidant by scavenging hydroxy radicals, hypochlorous acid, and singlet oxygen in multiple systems such as organic solvents, as well as under physiological conditions. It is suggested that this antioxidant activity is due to the strained conformation of the 5-membered dithiolane ring in the intramolecular disulfide form of LA. 

In biological systems LA is reduced to DHLA, which has been proved to be a more potent antioxidant. During transferring acyl groups bound to it from one part of the enzyme complex to another, LA is reduced to DHLA and again oxidized back to LA by lipoamide dehydrogenase forming NADH, and thus LA and DHLA act as redox pair by carrying electrons to NAD^+^ from the substrate of the dehydrogenase [18, 38]. DHLA has stronger antioxidant activity due to the two S-H groups and is readily oxidized back to 1,2-thiolanes. It acts readily as a potent scavenger of reactive oxygen species such as hydroxyl radicals, hypochlorous acid, superoxide anion radicals, and peroxyl radicals. It has also been suggested that DHLA may act as a prooxidant by the reduction of transition metals [38, 39]. 

In our study DHLA showed lower DPPH^●^ scavenging capacity than DODHLA. This discrepancy may be due to differences of the purity of these compounds as described later. The EC_50_ values reported for antioxidant compounds common in foods [29] were compared with the EC_50_ values in this study (Table 1). The free radical scavenging capacity observed for DODHLA indicates a similar capacity to that of *α*-tocopherol, which is a fat-soluble antioxidant. In addition to the structural conformation, the interaction between the potential antioxidant and DPPH^●^ can be influenced by the polarity of the solvent, as well as the affinity of the tested compound towards the solvent [29]. Although it is expected that one should at least obtain very similar EC_50_ values for DHLA and DODHLA, in our study lower EC_50_ value was observed for DHLA. This minor difference is probably due to the fact that the commercial DHLA was not extremely pure, which was detected when it was checked by TLC in our laboratory. Looking at the close similarity in the DPPH free radical scavenging behaviour between the two pairs LA/DHLA and DOLP/DODHLA, it is reasonable to speculate that this pair would exhibit a similar behaviour in biological systems. 

The mass spectral data observed for DO are completely agreeable with the previous data for DAG analyzed by APCI [40]. Since the hybrid molecules DOLA and DODHLA are synthesized for the first time in this study, there is no literature to compare with their mass spectral data. The difference observed in the mass for protonated molecular ions of DOLA and DODHLA was corresponding to *m/z* 2 units confirming the reduction of disulfide bond in the LA which is given in literature as 2.02 Da [41]. Major fragment ion observed was at *m/z* 529 [M–R_1_COOH+H]^+^ where one oleic acid moiety was dissociated from the molecule. The two fragments at *m/z* 527 and *m/z* 529 were observed for corresponding DAG ion in DOLA and DODHLA, which further highlighted the reduction of disulfide bond in the LA moiety. The common fragment ion at *m/z* 603 showed dissociation of DHLA moiety from DODHLA, leaving DAG ion with two oleic acids. The fragment at *m/z* 793 suggests an ion [M–H_2_O+H]^+^. However, the ion fragment at *m/z* 626 in DOLA and at *m/z* 628 in DODHLA may be due to some adduct formation in the mass analyser. Less fragmentation observed in APCI can also be attributed by neutral molecules and fatty acid or carboxylic acid molecules formed during ionisation because they are not visible in the mass spectrum.

The molecular ions and fragment ions at *m/z* 171 and 173 for LA and DHLA, respectively, as found in this study were in agreement with earlier findings [42]. The formation of [M–2H–H]^−^ ion at *m/z* 205 was also observed, probably due to dehydrogenation of dithiol group in [M–H]^−^ ion at high temperature, as it was suggested for methyl lipoate and methyl dihydro lipoate analysed by using GC-MS [43]. It is to be mentioned here that mass spectrum of DODHLA also showed an ion at *m/z* 809 but at very low intensity. To check the origin of these ions, we analysed newly synthesized both DOLA and DODHLA by HPLC-MS, and it was found that these compounds were separated from each other and still DODHLA contained both the ions at *m/z* 809 (at a very low abundance) and at *m/z* 811 as the major ion. Further, it showed that the ion at *m/z* 809 also appears at the same retention time as at *m/z* 811 while tracing these ions at selective ion mode suggesting some possibility of dehydrogenation of DODHLA into DOLP during the ionization in the HPLC-MS interphase (results are not shown). We performed HPLC-MS analyses of DODHLA at different vaporizer temperatures in MS 250, 300, 350, 400°C while maintaining same drying gas temperature at 350°C. Similar results were observed for area ratio when the drying gas temperature was constant at 300°C and vaporizer temperatures were changed from 250 to 400°C. Considering the results on the lowest peak area ratio between *m/z* at 811 and 809 and the noise level, it was decided to use 300°C as the vaporizer temperature and 350°C as the drying gas temperature in our method. 

DPPH test provides evidence of a potential free radical scavenging capacity; however, DPPH^●^ is not similar to ROS which occur naturally in biological systems. Further studies are planned to evaluate the antiobesity and overweight and *in vitro* and *in vivo* antioxidant properties of these hybrid compounds. The analysis using APCI provided soft fragmentation, yet informative mass spectra with protonated molecular ions and several fragment ions of the novel compounds DOLA and DODHLA. These data would be useful in identification and quantification of these conjugates and possible metabolites in future *in vitro* and *in vivo* studies. 

## Figures and Tables

**Table 1:** Free radical scavenging activity of DHLA and DODHLA.

Compound	Efficient concentration (EC_50_)^a^ **± **SD	Antradical power (ARP)^b^ **± **SD	No. moles DPPH reduced/mole of antioxidant **± **SD
DHLA	0.39 ± 0.04	2.56 ± 0.24	1.28 ± 0.12
DODHLA	0.22 ± 0.03	4.55 ± 0.55	2.27 ± 0.27

DHLA: dihydrolipoic acid; DODHLA: 1,3-dioleoyl-2dihydrolipoyl-*sn*-glycerol.

^a^Efficient Concentration = EC_50_.

^b^ARP value was calculated from 1/ EC_50_, the larger the ARP the more efficient the antioxidant **(**see [29]).

**Table 2:** Linear formula of LA, DHLA, DOLA, and DODHLA, retention time (RT, minutes), mono isotopic mass and HPLC-MS-APCI data.

Compound	RT (min)	Monoisotopic mass	Ions (*m/z*)	Suggested molecular ions/fragments	Relative intensity of ions (%)
LA^a^ [C_8_H_14_O_2_S_2_]	3.47	206.04	205 [M–H]^−^	[M–H]^−^	100
			171 [M–34–H]^−^	[M–H_2_S–H]^−^	6
			411 [2M–H]^−^	[2M–H]^−^	6
DHLA^b^ [C_8_H_16_O_2_S_2_]	3.46	208.06	207 [M–H]^−^	[M–H]^−^	72
			173 [M–34–H]^−^	[M–H_2_S–H]^−^	3
			205 [M–2H–H]^−^	[M–2H–H]^−^	100
			171 [M–2H–34–H]^−^	[M–2H–H]^−^	15
DO^c^ [C_39_H_72_O_5_]	15.13	620.54	621 [M+H]^+^	[M+H]^+^	3
			603 [M–18+H]^+^	[M–H_2_O+H]^+^	100
			339 [M–282+H]^+^	[M–R_1_COOH+H]^+^	80
DOLA^d^ [C_47_H_84_O_6_S_2_]	17.68	808.57	809 [M+H]^+^	[M+H]^+^	100
			603 [M–206+H]^+^	[M–R_2_COOH+H]^+^	2
			527 [M–282+H]^+^	[M–R_1_COOH+H]^+^	7
			339 [M–206–282+18+H]^+^	[M–R_1_COOH–R_2_COOH+H_2_O+H]^+^	3
			826 [M+17+H]^+^	Unidentified adduct	7
DODHLA^e^ [C_47_H_86_O_6_S_2_]	17.05	810.59	811 [M+H]^+^	[M+H]^+^	26
			793 [M–18+H]^+^	[M–H_2_O+H]^+^	15
			603 [M–208+H]^+^	[M–R_3_COOH+H]^+^	76
			529 [M–282+H]^+^	[M–R_1_COOH+H]^+^	100
			339 [M–208–282+18+H]^+^	[M–R_3_COOH–R_1_COOH+H_2_O+H]^+^	35
			828 [M+17+H]^+^	Unidentified adduct	13

^a^LA: *α*-lipoic acid; ^b^DHLA: dihydro lipoic acid; ^c^DO: 1,3-dioleoyl-*sn*-glycerol; ^d^DOLA: 1,3-dioleoyl-2-lipoyl-*sn*-glycerol; ^e^DODHLA: 1,3-dioleoyl-2Dihydrolipoyl-*sn*-glycerol;

*R_1_COOH = oleic acid general formula; R_2_COOH = *α*-lipoic acid general formula; R_3_COOH = dihydrolipoic acid general formula.

## Abbreviations

APCI: Atmospheric pressure chemical ionization
DAG: Diacyl-*sn*-glycerol
DHLA: Dihydrolipoic acid
DO: 1,3-Dioleoyl-*sn*-glycerol
DOLA: 1,3-Dioleoyl-2-lipoyl-*sn*-glycerol
DODHLA: 1,3-Dioleoyl-2-dihydrolipoyl-*sn*-glycerol
DMAP: 4-Dimethylaminopyridine
EDCI: 1-Ethyl-3-(3-dimethylaminopropyl) carbodiimide hydrochloride
HPLC: High-performance liquid chromatography
LA: *α*-Lipoic acid
MAG: Monoacyl-*sn*-glycerol
MS: Mass spectrometry
NMR: Nuclear magnetic resonance
PMA: Phosphomolybdic acid
RP: Reversed phase
TAG: Triacyl-*sn*-glycerol
TLC: Thin-layer-chromatography
TMS: Tetramethylsilane.
